# Immunoprecipitation of Amyloid Fibrils by the Use of an Antibody that Recognizes a Generic Epitope Common to Amyloid Fibrils

**DOI:** 10.1371/journal.pone.0105433

**Published:** 2014-08-21

**Authors:** Erin R. Greiner, Jeffery W. Kelly, Fernando L. Palhano

**Affiliations:** 1 Departments of Chemistry and Molecular and Experimental Medicine and the Skaggs Institute for Chemical Biology, The Scripps Research Institute, La Jolla, California, United States of America; 2 Instituto de Bioquímica Médica Leopoldo de Meis, Programa de Biologia Estrutural, Universidade Federal do Rio de Janeiro, Rio de Janeiro, Brazil; Universitat Autònoma de Barcelona, Spain

## Abstract

Amyloid fibrils are associated with many maladies, including Alzheimer’s disease (AD). The isolation of amyloids from natural materials is very challenging because the extreme structural stability of amyloid fibrils makes it difficult to apply conventional protein science protocols to their purification. A protocol to isolate and detect amyloids is desired for the diagnosis of amyloid diseases and for the identification of new functional amyloids. Our aim was to develop a protocol to purify amyloid from organisms, based on the particular characteristics of the amyloid fold, such as its resistance to proteolysis and its capacity to be recognized by specific conformational antibodies. We used a two-step strategy with proteolytic digestion as the first step followed by immunoprecipitation using the amyloid conformational antibody LOC. We tested the efficacy of this method using as models amyloid fibrils produced *in vitro*, tissue extracts from *C. elegans* that overexpress Aβ peptide, and cerebrospinal fluid (CSF) from patients diagnosed with AD. We were able to immunoprecipitate Aβ_1–40_ amyloid fibrils, produced *in vitro* and then added to complex biological extracts, but not α-synuclein and gelsolin fibrils. This method was useful for isolating amyloid fibrils from tissue homogenates from a *C. elegans* AD model, especially from aged worms. Although we were able to capture picogram quantities of Aβ_1–40_ amyloid fibrils produced *in vitro* when added to complex biological solutions, we could not detect any Aβ amyloid aggregates in CSF from AD patients. Our results show that although immunoprecipitation using the LOC antibody is useful for isolating Aβ_1–40_ amyloid fibrils, it fails to capture fibrils of other amyloidogenic proteins, such as α-synuclein and gelsolin. Additional research might be needed to improve the affinity of these amyloid conformational antibodies for an array of amyloid fibrils without compromising their selectivity before application of this protocol to the isolation of amyloids.

## Introduction

Maintenance of protein homeostasis, or proteostasis, is accomplished by the proteostasis network comprising biological pathways that control the rate of protein synthesis and the efficiency of protein folding, trafficking and degradation [1]. The aggregation of peptides or proteins, exacerbated by aging, is genetically and pathologically linked to degenerative disorders, including Alzheimer’s disease (AD), Parkinson’s disease, and the systemic amyloid diseases [2]. A wide range of proteins, including those normally existing in a soluble folded state or as an intrinsically disordered monomer, can form cross-β-sheet amyloid fibrils owing to a mutation or because of environmental alterations [3]. Amyloid deposits can be detected using Congo red birefringence or thioflavin T fluorescence, and are often associated with glycosaminoglycans, the amyloid P component, or other proteins [3]. Amyloid fibrils are made up of multiple interacting filaments, which are each comprised of thousands of monomers arranged at least as two-layer cross-β-sheets [4]. Amyloid is generally relatively resistant to denaturation and proteolysis [5]. Because amyloid is stabilized by backbone H-bonding and side chain-side chain hydrophobic interactions, it has been proposed that any protein, regardless of its amino acid sequence, can form amyloid fibrils if subjected to appropriate solution conditions [6], [7].

Since the amyloid fibrils from different sources display common characteristics, several groups have developed antibodies capable of recognizing the so-called ‘universal amyloid epitope’ [8]–[11]. All of these antibodies are able to distinguish between the mature amyloid structure and the monomeric or oligomeric intermediate precursors of amyloid aggregation [8]–[10]. These antibodies can be important tools to disrupt amyloid fibrils, in detection of amyloid disease related aggregates, and to isolate amyloid fibrils from complex solutions [8]–[10]. Among the amyloid conformational antibodies developed, LOC, originally produced by Glabe’s group [10], is a commercially available rabbit polyclonal antibody raised against mature amyloid fibrils derived from islet amyloid polypeptide (IAPP). This antibody can distinguish between Aβ amyloid fibrils and Aβ in oligomeric and monomeric states [10].

A specific and sensitive protocol to isolate and detect amyloids is much in need for the diagnosis of amyloid diseases. For example, the current methodologies require Congo red staining of biopsies, a method with low specificity and sensibility [12], [13] that is a mandatory criterion for inclusion in clinical trials for peripheral amyloidosis [14]. Also, a protocol to isolate amyloid fibrils would be useful for the discovery of new amyloids. In addition to the association of amyloid fibrils with several pathologies, proteins that self-assemble into amyloid can also serve specific biological functions [15]–[17]. These functional amyloid fibrils are used by organisms to perform diverse physiological functions such as biofilm formation [18], [19], cell adhesion [20], synaptic remodeling and learning [21], template to melanin biosynthesis [22], [23], and peptide hormones [24]. Other examples include mitochondrial protein MAVS [25], the necrosome proteins RIP1/RIP3 [26], and several dozen of proteins involved in RNA granule formation [27].

In this work, we took advantage of the unique physical chemical properties of amyloid fibrils in order to develop a method designed to isolate amyloid fibrils from complex biological solutions such as cell lysate from a multi-cellular organism. For this purpose, we used amyloid fibrils produced *in vitro* from three different proteins, namely Aβ_1–40_, α-synuclein (α-syn) and gelsolin, as well as the lysate of wild type and an AD model of *Caenorhabditis elegans* (*C. elegans*) worms. Here we show that amyloid fibrils from all three proteins tested maintained their amyloid architecture after incubation with the proteolytic enzyme proteinase K (PK) and after incubation with the organic solvent acetone. After PK digestion and acetone precipitation, we immunoprecipitated the amyloid fibrils using the fibril-specific, conformation-dependent antibody LOC [10]. This strategy was successful for capturing Aβ_1–40_ amyloid fibrils but failed to capture α-synuclein (α-syn) and gelsolin fibrils. This result was consistent with the ability of LOC antibody to recognize these three amyloid fibrils when assayed by dot blot. We applied this method to the lysates from the AD worm model CL2006 [28] in which overexpressed human Aβ peptide aggregates as amyloid fibrils_._ We immunoprecipitated more Aβ fibrils in the older worms (day 8) when compared with young worms (days 1 and 5), validating our strategy in a biological system. Since the immunoprecipitation (IP) method was sensitive enough to capture and detect picograms of Aβ amyloid fibrils produced *in vitro*, we searched for Aβ aggregates in cerebrospinal fluid of patients diagnosed with AD, but we could not detect any aggregates. We discuss the limitations and potential applications of this method.

## Materials and Methods

### Preparation of amyloid fibrils

Aβ_1–40_ [29], α-syn [30] and the 8 kDa gelsolin fragment (residues 173–242) [31] were purified as previously described. Aβ_1–40_ at 216 µg/ml was aggregated in 50 mM sodium phosphate buffer, pH 7.4, 150 mM NaCl, 0.02% NaN_3_ at 37°C for 7 days with agitation. α-syn at 1,960 µg/ml was incubated in 10 mM Tris buffer pH 7.4, 100 mM NaCl, 0.02% NaN_3_ under the same conditions described for Aβ_1–40_. The 8 kDa gelsolin fragment peptide was incubated at 60 µg/ml in 50 mM sodium phosphate buffer, pH 6.8, 100 mM NaCl, 0.02% NaN_3_ at 37°C with agitation for 24 h. The sample was then centrifuged (16,000 g for 15 min at 4°C) and the pellet was resuspended in 50 mM sodium phosphate buffer, pH 7.4, 150 mM NaCl, 0.02% NaN_3_ to obtain a final concentration of 600 µg/ml.

### Congo red and thioflavin T binding assays

Fibril formation was assessed using Congo red and thioflavin-T (ThT) binding assays. For Congo red binding, the samples were diluted to a final concentration of 65 µg/ml in 5 mM potassium phosphate and 150 mM NaCl at pH 7.4 containing 10 µM Congo red and absorbance was recorded at 540 and 477 nm [32]. For ThT binding assays, the samples were diluted to 65 µg/mL in 5 mM potassium phosphate and 150 mM NaCl at pH 7.4 containing 20 µM ThT and binding was monitored using a spectrofluorimeter to measure the fluorescence increase (excitation at 450 nm and fluorescence emission at 465–520 nm) [33].

### *In vitro* kinetic proteinase K assay

Aβ_1–40,_ α-syn or gelsolin fibrils (65 µg/ml) were incubated with 0.13 µg/ml (1∶500 w/w) proteinase K (Roche) in phosphate buffer (50 mM sodium phosphate, pH 7.4, 150 mM NaCl) containing ThT (20 µM) at 42°C. Every 10 min, the plates were shaken for 5 s, and fluorescence (excitation at 440 nm, emission at 485 nm) was monitored using a Spectra Gemini EM fluorescence plate reader.

### Dot blot assay

Samples of Aβ_1–40,_ α-syn or gelsolin (65 µg/ml) (monomeric or fibrillar; PK-digested (0.13 µg/ml for 2 h at 42°C) or not) were spotted (2 µl) onto nitrocellulose membrane. The membrane was blocked using 1 vol PBS + 1 vol blocking solution (Odyssey) for 1 h. The membrane was incubated with LOC antibody (1∶1,000, Millipore) diluted in 1 vol TBST (50 mM Tris pH 7.6, 0.9% NaCl, 0.1% Tween 20) +1 vol blocking solution for 1 h, washed 3 times with TBST and then incubated for 1 h with goat anti-rabbit secondary antibody conjugated to IRDye 680 CW (1∶5,000) and developed/quantified using an Odyssey Infrared Imaging System.

### Western blotting

The samples were boiled for 15 min in the presence of Laemmli buffer + 4 M urea in order to monomerize the fibrils. SDS-PAGE was performed under reducing conditions using 16% tris-tricine gels. Samples were transferred to nitrocellulose membranes and probed with 6E10 antibody (1∶10,000) for Aβ_1–40_, syn-1 antibody (1∶10,000) for α-syn and monoclonal anti α tubulin (1∶10,000) for tubulin. For gelsolin, a rabbit polyclonal antibody (1∶10,000) developed by Balch’s group [34] was used. Blots were then probed with goat anti-mouse secondary antibody conjugated to IRDye 800 CW (1∶10,000) for Aβ_1–40_ fibrils, α-syn and tubulin and goat anti-rabbit secondary antibody conjugated to IRDye 680 CW (1∶10,000) for gelsolin and developed/quantified using an Odyssey Infrared Imaging System.

### Electron Microscopy

The samples were prepared as described by Azevedo and colleagues [35].

### Protein quantification

Total protein concentrations were determined using the Pierce BCA assay according with manufacturers’ instructions (Pierce).

### Preparation of *C. elegans* extracts

CL2006 (dvIs2[*unc-54p::Aβ_1–42_*+ *rol-6*]) [28] and the wild-type strain N2 (Bristol) were obtained from the *Caenorhabditis* Genetics Center (University of Minnesota, Minneapolis, MN). Synchronized eggs were harvested by bleaching and worms were grown in liquid culture containing fluorodeoxyuridine (FUDR; 0.12 mM; Sigma) and OP50 bacteria (5 mg/mL), as previously described [36]. Worms were maintained at 20°C, aged until day 1, day 5, and day 8 of adulthood, then washed three times in M9 buffer and flash frozen in liquid nitrogen prior to western blot analysis. Crude extracts were prepared in PBS buffer, 1% Triton X100 with 1X Proteinase inhibitor Cocktail (PIC, Roche) using the Precellys 24 homogenizer (Peqlab) and ceramic beads (2.8 mm diameter) [37] and centrifuged at 700 g for 3 min at 4°C to obtain post debris supernatant (PDS), as previously described [38].

### Cerebrospinal fluid (CSF)

Human CSF was purchased from Biochemed Services, Winchester, VA and stored at −80°C until use. We used three independent samples from different patients diagnosed with Alzheimer’s disease.

### Isolation of amyloid fibrils

Three hundred microliters of worm PDS (N2 or CL2006) (50–500 µg/ml final protein concentration) or human CSF (100–500 µg/ml final protein concentration) diluted in PBS pH 7.4 with 0.1% Tween 20 in the absence or in the presence of differing amounts of amyloid fibrils were sonicated for 15 min in a Fisher Scientific FS60 Sonic Cleaner at 4°C. The samples were digested with proteinase K (1∶500) for 2 h at 42°C. Then, one volume of cold acetone was added to each sample and the samples were centrifuged at 16,000 g for 10 min at 4°C. The pellet was resuspended in 300 µl of PBS with 0.1% Tween 20 with 1X protease inhibitor cocktail (Roche). The samples were sonicated for 5 min 4°C and 5 µl of LOC (Millipore) antibody was added and the samples were incubated for 24 h at 4°C with agitation. The bead slurry was added (60 µl, East Coast Bio Protein G gel Slurry) and the samples were incubated for additional 24 h at 4°C with agitation. After washing 3 times with 300 µl PBS, the samples were eluted from the beads with 30 µl glycine pH 2.3 at 65°C for 10 min plus 5 min sonication. For all the steps involving the centrifugation/washing of the beads, the samples were centrifuged at 78 g for 1 min at 4°C.

## Results and Discussion

**Figure 1:**
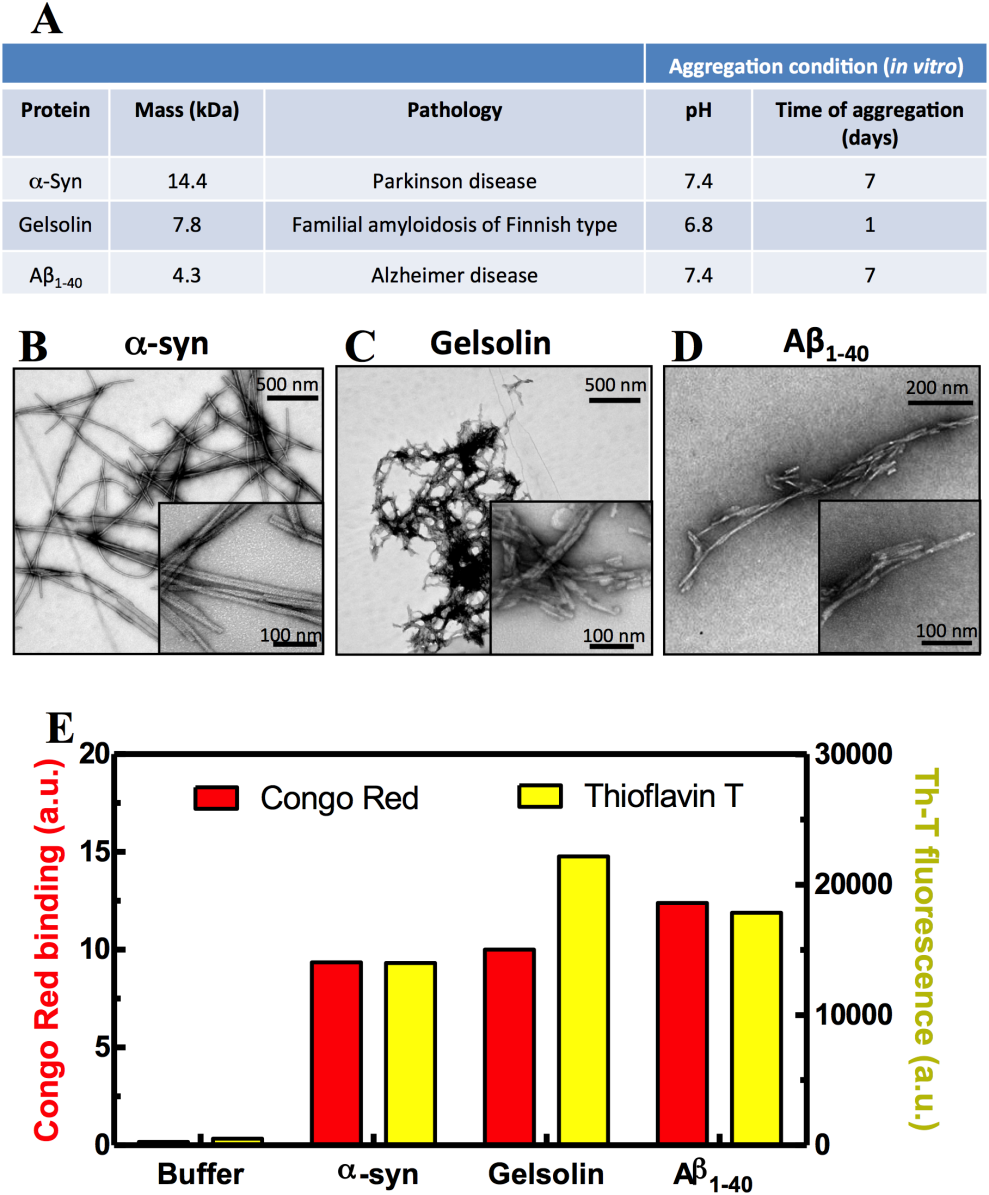
Characterizing the morphological and tinctorial properties of the aggregates. (A) Table describing the peptides and aggregation conditions used in this study. (B–D) Transmission Electron Microscopy (TEM) images showing the morphology of the aggregates used in this study: (B) α-synuclein fibrils (α-syn), (C) gelsolin fibrils, and (D) Aβ_1–40_ fibrils. (E) Congo Red (CR, red bars) and Thioflavin T (ThT, yellow bars) bound to the aggregates shown in B–D. All the samples were used at 65 µg/ml and CR and ThT were used at 10 µM and 20 µM, respectively. The buffer for the CR and ThT binding assays was 5 mM potassium phosphate and 150 mM NaCl at pH 7.4. For ThT: Ex = 450 nm and Em = 465–520 nm. For CR: absorbance at 540/447 nm.

We produced amyloid fibrils using three different proteins, namely, Aβ_1–40_, α-syn and the 8 kDa fragment of gelsolin. Aβ_1–40_ is the peptide associated with Alzheimer’s disease [39], α-syn is associated with Parkinson’s disease [40], and gelsolin with Familial amyloidosis of Finnish type [41], [42] (Figure 1A). The fibrils formed from the three different proteins presented with the typical amyloid structure (panels B–D), as seen by transmission electron microscopy (TEM). The aggregates formed by α-syn were long and twisted (Figure 1B), whereas gelsolin and Aβ_1–40_ aggregates were shorter, with some clusters (Figure 1C, 1D). These results were confirmed by Congo Red (CR; Figure 1E, red bars) and ThT (Figure 1E, yellow bars) binding. These two compounds are amyloid-specific dyes that change their spectroscopic behavior when bound to the cross-β fold present in amyloid fibrils [32], [33]. As a negative control, we assessed ThT and CR binding using buffer alone (Figure 1E) or soluble peptides of Aβ_1–40,_ α-syn or gelsolin (not shown due to the similarity with the buffer control). On average, we observed a 10–20 fold higher ThT and CR signal with aggregated peptides than with buffer alone (Figure 1E).

**Figure 2:**
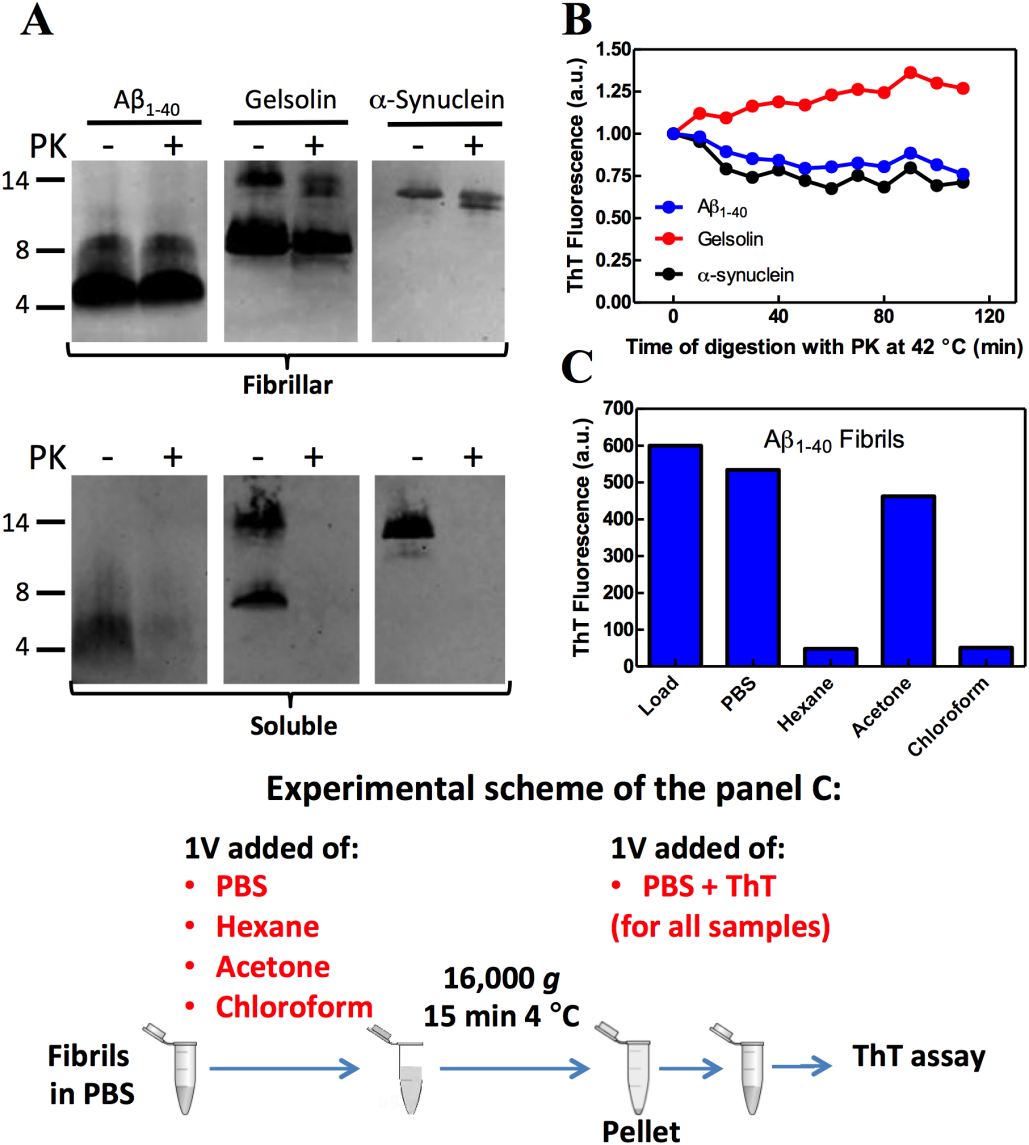
Amyloid fibrils maintained their amyloid architecture after proteolytic digestion and acetone extraction. (A) Aβ_1–40,_ α-syn or gelsolin peptides (65 µg/ml) in a fibrillar (upper gel) or soluble (lower gel) state were incubated in the absence or presence of 0.13 µg/ml (1∶500, w/w) proteinase K (PK) for 2 h at 42°C. The digestion was conducted in 50 mM sodium phosphate, pH 7.4, 150 mM NaCl buffer. The reaction was stopped by boiling the samples in Laemmli buffer with 2% SDS and the samples were resolved by 16% SDS-PAGE. Western blot using 6E10 (Aβ_1–40_), syn-1 (α-syn) or a gelsolin-specific antibody is presented. (B) The same reaction described in panel A was performed in the presence of 20 µM of thioflavin T (ThT) and the florescence was monitored every 10 min. Ex = 440 nm and Em = 485 nm. (C) Aβ_1–40_ amyloid fibrils at 65 µg/ml concentration were diluted in 1 volume (1 V) of PBS, hexane, acetone or chloroform and centrifuged (16,000 g) for 10 min at 4°C. The pellet was resuspended in phosphate buffer with 20 µM ThT and the fluorescence measured. An aliquot of undiluted/uncentrifuged fibrils was used as the load. Ex = 450 nm and Em = 465–520 nm.

Next we compared the susceptibility of amyloid fibrils versus soluble peptides to the proteolytic enzyme, proteinase K (PK), a serine endopeptidase with a broad spectrum of action [43]. We used PK digestion to reduce the molecular complexity of the proteome while preserving the fibrillar amyloid architecture. For this purpose, we incubated the Aβ_1–40_, α-syn and gelsolin peptides, either as soluble peptides or in an aggregated state, in the presence of PK (500∶1, w/w) for 2 h at 42°C and then assessed the stability of the peptides using Western blot with peptide-specific antibodies. As expected [44], only the fibrillar material was resistant to PK digestion (Figure 2A). Similar results were observed using silver stained gels, thus excluding the possibility that PK digestion destroyed the epitopes that the peptide-specific antibodies recognize (data not shown). A plausible explanation for the bands with higher molecular weight in Figure 2A for gelsolin and Aβ is that some dimers could be resistant even to prolonged boiling in the presence of 2% SDS. The results shown in Figure 2A are from a PK digestion performed in saline phosphate buffer, but similar results were observed when the digestion was carried out in a more complex solution such as a tissue homogenate from wild type *C. elegans* (not shown). To determine whether the fibrils are indeed amyloid after PK digestion, we monitored ThT fluorescence as a function of time to assess the integrity of the Aβ_1–40_, α-syn and gelsolin fibrils in the presence of PK (Figure 2B). ThT fluorescence of all the amyloids tested herein was generally unchanged during the PK digestion (Figure 2B), confirming that the PK-resistant fibrils largely retained their amyloid structure.

Cell lysates are complex mixtures of proteins, lipids, carbohydrates, and nucleic acids, and these molecules can interfere with and compromise the purification of amyloid fibrils. Thus, we searched for a second step after PK digestion that could be used to reduce the complexity of the lysate. Since lipids are the second most abundant macromolecule in cellular lysates [45], we incubated the fibrils with several organic solvents well known to solubilize lipids. After incubation of Aβ_1–40_ fibrils dissolved in PBS with 1 volume of the organic solvent, we centrifuged the samples and resuspended the pellet in a new solution of PBS containing ThT and measured ThT fluorescence (Experimental scheme at the bottom of Figure 2). The only solvent tested that did not disrupt the Aβ_1–40_ fibrillar architecture was acetone (Figure 2C). Acetone is usually used to solubilize non polar lipids, which in the case of *C. elegans* tissue homogenates accounts for about 20% of the dry body mass [46]. Similar results were obtained using α-syn and gelsolin fibrils (not shown).

**Figure 3:**
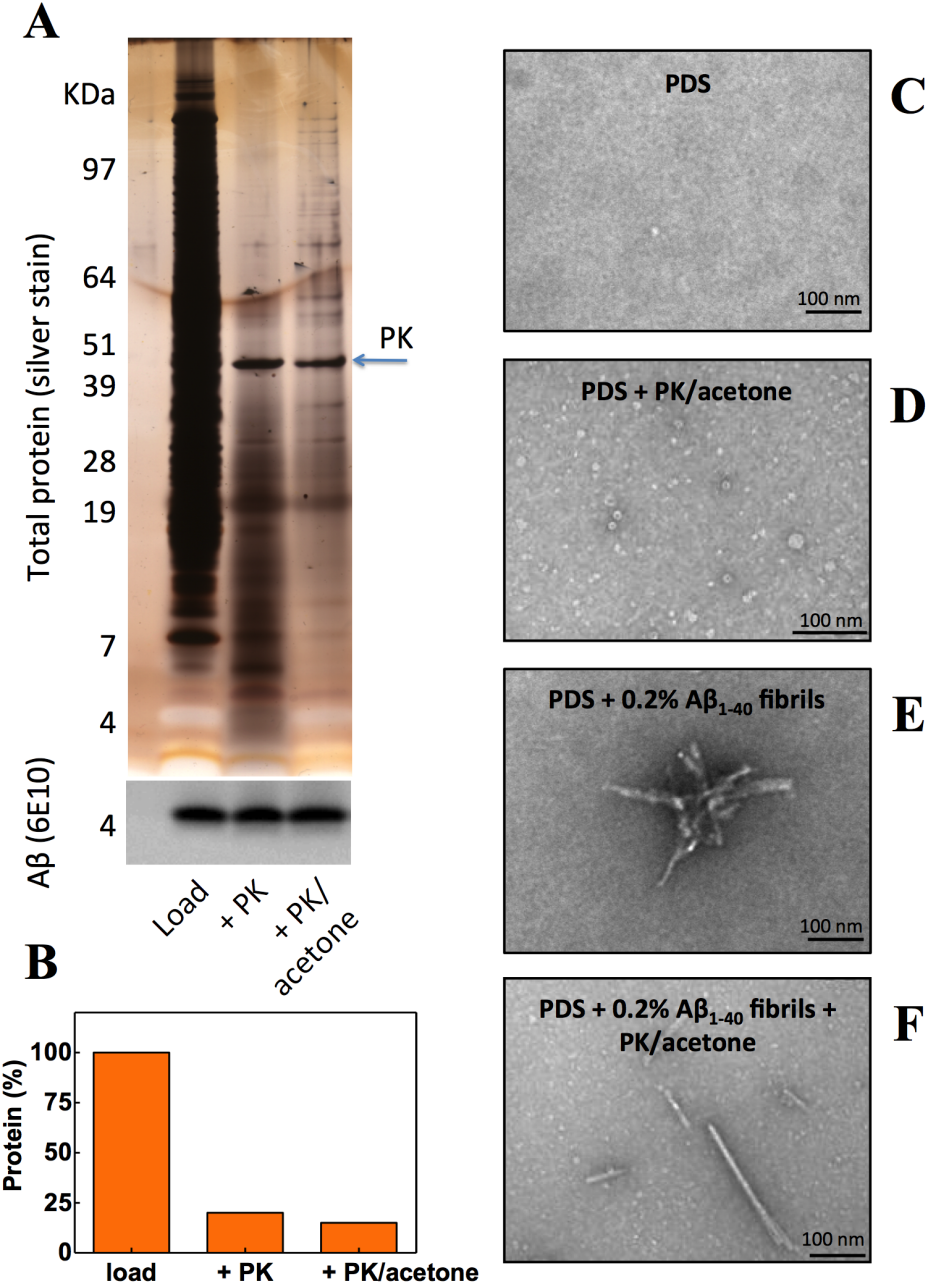
Effect of proteinase K digestion and acetone precipitation on the protein content of a complex biological extract. (A and B) The complex biological extract was obtained by mechanical disruption of wild type *C. elegans* worms followed by a brief centrifugation (700 g for 3 min) to remove unlysed worms. Aβ_1–40_ fibrils (0.2% w/w protein concentration) were added to the worm post debris supernatant (PDS) and the samples were digested with PK (1∶500) for 2 h at 42°C followed by acetone precipitation. An aliquot before PK digestion (load), after PK digestion (+ PK) and after PK digestion and acetone precipitation (+ PK/acetone) were resolved by SDS-PAGE (A) or the protein was quantified by BCA assay (B). In the panel A, the upper gel is silver stained and the lower gel is a Western blot for Aβ using the 6E10 antibody. (C–F) TEM images of PDS. PDS was incubated in the absence (C) or in the presence of 0.2% Aβ_1–40_ fibrils (E) before the PK/acetone step. PDS incubated in the absence (D) or in the presence of 0.2% Aβ_1–40_ fibrils (F) was digested with PK and precipitated with acetone. Note that amyloid fibrils are present only in the samples to which Aβ_1–40_ fibrils were added (E and F).

Having demonstrated that the amyloid fibrils tested herein were resistant to PK digestion and incubation with acetone, we asked what effect these treatments would have on the proteome of a complex multi-cellular lysate. The complex lysate was obtained by mechanical lysis of the wild type N2 strain of *C. elegans*, followed by centrifugation (700 g for 3 min) to obtain post debris supernatant (PDS). We spiked the PDS with a small amount of Aβ_1–40_ amyloid fibrils (0.2%, w/w protein) and then digested the lysate with PK and precipitated the PK-digested lysate with 1 volume of acetone. As visualized by silver-stained SDS-PAGE (Figure 3A, upper gel) and quantified by BCA (Figure 3B), the amount of protein remaining after PK digestion and acetone extraction decreased by about 80–85%. Nevertheless, the amount of Aβ_1–40_ recovered from the treated lysates was unaffected by these harsh conditions (Figure 3A, lower gel). Interestingly, treatment of the PDS alone with PK and acetone resulted in the production of annular aggregates, similar to those described during the aggregation of amyloidogenic proteins (compare Figure 3C with Figure 3D) [47], [48]. Amyloid fibrils were observed only in the samples that were spiked with Aβ_1–40_ (compare Figure 3D with 3F) and the synthetic fibrils maintained their fibrillar structure after treatment with PK and acetone (compare Figure 3E with Figure 3F). Similar results were obtained using α-syn and gelsolin fibrils (not shown). Assuming that functional amyloid exists in *C. elegans* and is resistant to PK digestion, our inability to detect any fibrils of functional amyloid may reflect the low abundance of functional amyloid, probably less than 0.2% of the total proteome. Another possibility is that the putative functional amyloids of *C. elegans* are more susceptible to PK digestion when compared to synthetic fibrils. In fact, functional amyloids described recently by McKnight’s group were dynamic and highly sensitive; that is, those fibrils were easily denatured by SDS treatment [27]. It is important to note that the experiment described in the Figure 3 was performed using PDS from worms at day 1 of adulthood. There is some evidence that endogenous amyloid exists in aged worms [49], thus in the future, aged worms may be interesting models for the identification of functional amyloids.

**Figure 4:**
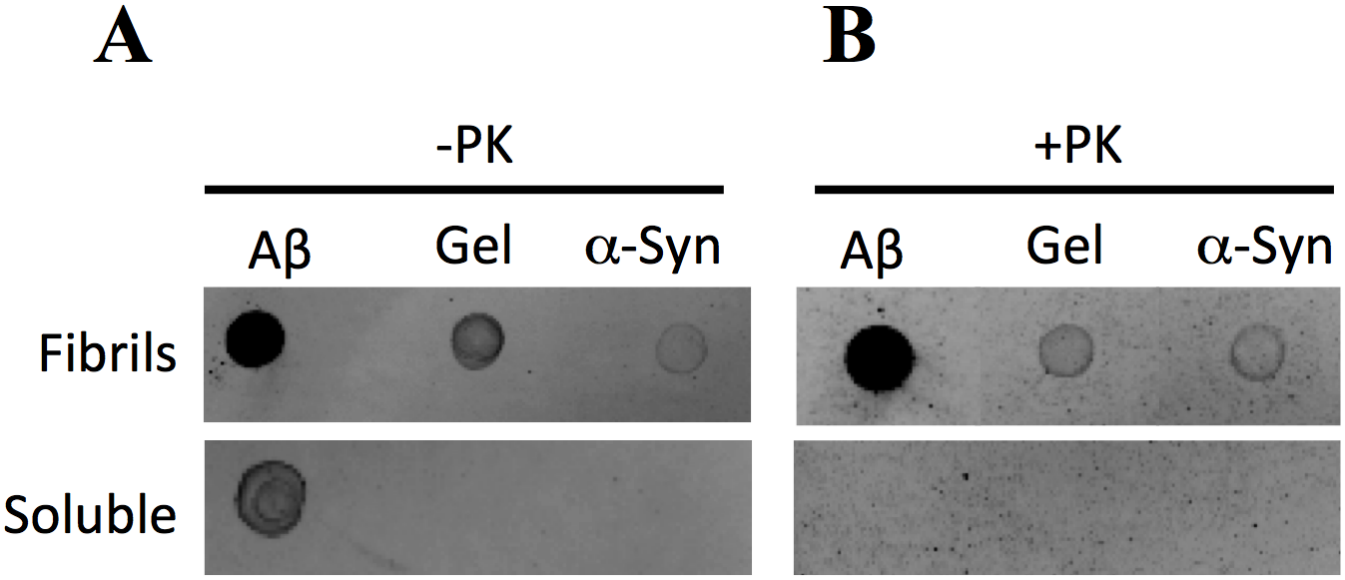
Dot blot using the LOC antibody that recognizes a generic amyloid epitope. Aβ_1–40,_ α-syn or gelsolin peptides (65 µg/ml) in a fibrillar or soluble state were incubated in the absence (A) or in the presence (B) of PK (1∶500) for 2 h at 42°C then spotted onto nitrocellulose membrane loaded with LOC antibody.

Molecular tools that are highly specific and sensitive to structural motifs that are present in all amyloid fibrils would be useful for the purpose of isolating amyloid fibrils present in complex solutions [50], [51]. In this context, several groups have developed antibodies that recognize an universal amyloid epitope [8]–[10]. Examples of these include the WO1 and WO2 antibodies from Wetzel’s group [8] and the B10 and B10AP antibodies from Fandrich’s lab [9], produced using amyloid fibrils derived from Aβ_1–40_ or Aβ_1–42,_ and OC and LOC from Glabe’s group [10], produced using amyloid fibrils from Aβ_1–42_ and IAPP peptides, respectively. All these antibodies are able to distinguish between mature amyloid fibrils and monomeric or oligomeric species. Moreover, these antibodies recognized amyloid fibrils produced from several proteins, such as Aβ_1–40_, Aβ_1–42_, α-syn, IAPP, yeast prions, polyglutamine, and transthyretin, among others, confirming the existence of a common universal amyloid epitope [8]–[10], [52]. We chose to use the LOC antibody for immunoprecipitation because this antibody is commercially available and was produced using IAPP, reducing the possibility that the antibody specifically recognizes one of the peptide used in this study (Aβ_1–40_, α-syn and gelsolin) instead of the conformational amyloid epitope. It is important to note that the primary amino acid sequence of Aβ_1–40_ and IAPP have 23% identity and 38% similarity, rendering the LOC antibody more efficacious for Aβ than α-syn and gelsolin, as will be discussed here. To probe the efficacy of the LOC antibody, we performed a dot blot assay using the amyloid fibrils described in Figure 1 as well as soluble peptides from Aβ_1–40_, α-syn, and gelsolin. In order to compare the affinity of LOC for the different fibrils used, we spotted the same mass of each peptide onto the nitrocellulose membrane. We observed that LOC was able to bind to all of the amyloid fibrils tested (Figure 4A); however, the binding to Aβ_1–40_ fibrils was much stronger than to α-syn and gelsolin fibrils. We also detected binding to soluble Aβ_1–40_ peptide. This binding was less intense than the binding to Aβ_1–40_ fibrils but was stronger than that to α-syn and gelsolin fibrils. At the concentration used in this study, we did not detect any binding of LOC to soluble α-syn and gelsolin peptides. After the PK digestion, the LOC antibody still binds to the amyloid fibrils of Aβ_1–40_, α-syn and gelsolin to a similar extent to that observed with undigested fibrils (Figure 4B). Binding to soluble Aβ_1–40_ is no longer detectable, since the soluble peptide is totally degraded by PK (Figure 2A). Similar results were obtained with PK digestion followed by acetone precipitation (not shown). This result corroborates the electron microscopy results showing that Aβ_1–40_, α-syn and gelsolin amyloid fibrils maintained their amyloid structure, as well as the universal amyloid epitope, after PK digestion and acetone precipitation (Figure 3F).

**Figure 5:**
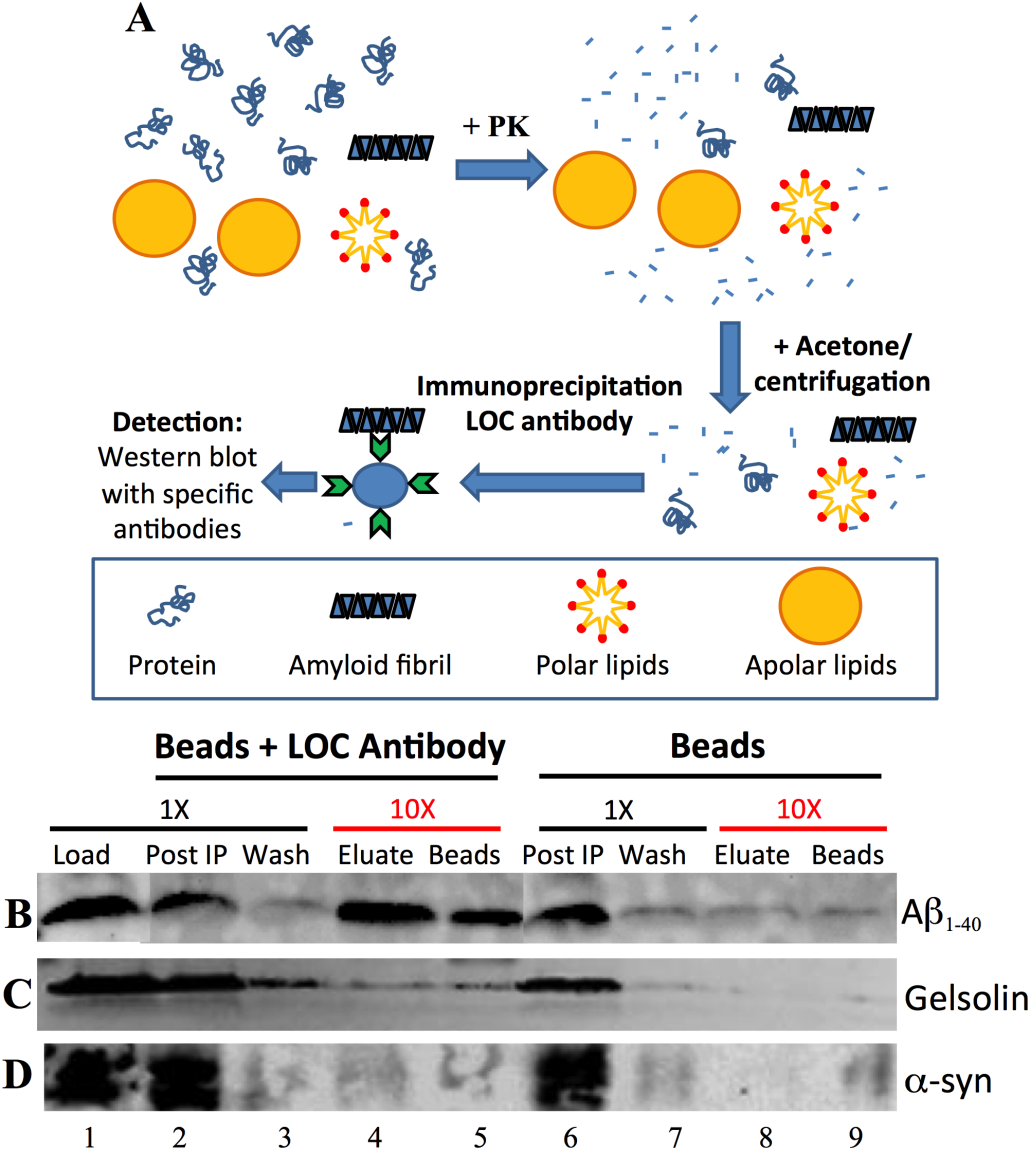
Immunoprecipitation of amyloid fibrils using the LOC antibody. (A) Schematic of the protocol used to isolate amyloid fibrils. (B) Aβ_1–40,_ (C) gelsolin or (D) α-syn amyloid fibrils (0.2% w/w) were added to worm PDS, digested with PK for 2 h at 42°C and precipitated with 1 volume of cold acetone. The pellet was resuspended in buffer containing LOC antibody and the IP was performed as described in Materials and Methods. As a negative control, we performed the IP in the absence of LOC antibody (beads). The samples were resolved by SDS-PAGE (16% tris-tricine gels) and probed for Aβ_1–40,_ gelsolin, or α-syn by western blotting.

To perform the immunoprecipitation (IP) of amyloid fibrils in a complex solution, we spiked a small amount (0.2% w/w protein) of amyloid fibrils produced *in vitro* (Figure 1) into the wild type worm PDS and followed the protocol described in the scheme of Figure 5A. The PDS + amyloid fibrils were digested with PK for 2 h at 42°C, precipitated with acetone and then immunoprecipitated with LOC antibody. Bound amyloid fibrils were then eluted using a combination of low pH and sonication. As a control, we used Protein G beads alone in the absence of LOC antibody. The fibrils immunoprecipitated by the LOC antibody were detected by SDS-PAGE followed by western blot using antibodies specific to monomeric Aβ_1–40,_ α-syn or gelsolin (See Materials and Methods). We observed that for Aβ_1–40_ fibrils a considerable amount of the protein was recovered in the eluate of the IP (Figure 5B, lane 4), suggesting that this method was able to purify amyloid fibrils from a complex solution. Not all the bound Aβ_1–40_ fibrils were recovered in the eluate since Aβ_1–40_ fibrils were detected after boiling the beads used in the IP (Figure 5B, lane 5). It is important to note that the amount of the sample applied in the SDS-PAGE for the eluate and beads fractions (Lanes 4, 5, 8 and 9, Figure 5) were 10 fold higher (10X) when compared with the other fractions (Load, Post IP and Wash in the lanes 1, 2, 3, 6 and 7-1X) (See details in the legend of Figure 5). We used this methodology in order to better detect any possible amyloid fibril immunoprecipitated by our protocol. No amyloid fibrils were recovered when the IP was performed in the absence of the LOC antibody (Figure 5B, lanes 8 and 9). For gelsolin and α-syn fibrils, we observed that most of the fibrils were present in the fraction that did not bind to the Protein G beads (Post IP) even when the LOC antibody was present, suggesting that the IP was inefficient (Figure 5C–D). Increasing the amount of gelsolin and α-syn fibrils added (0.5, 1 and 5%) or increasing the sonication time of the samples (60 min), in order to enhance the fibril fragmentation to enable a higher surface contact between the fibrils and the antibody, did not improve the efficacy of the IP for gelsolin and α-syn fibrils (data not shown). We also omitted the PK digestion and the acetone precipitation step but again we were unable to immunoprecipitate gelsolin and α-syn fibrils (data not shown).

**Figure 6:**
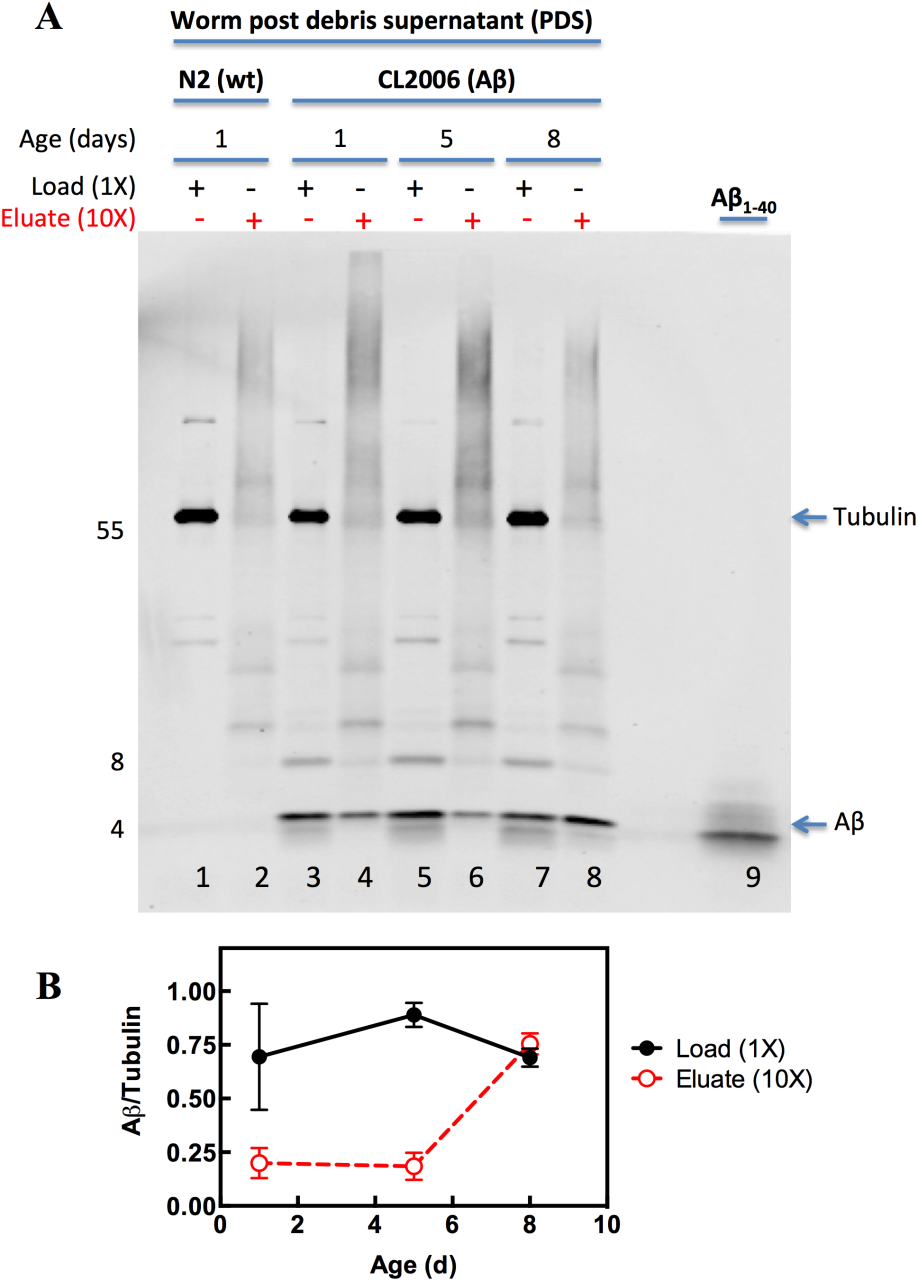
Immunoprecipitation of amyloid fibrils from tissue extracts of a *C. elegans* strain that overexpresses human Aβ_1–42_ peptide. (A) Worm post debris supernatant (PDS) from N2 (wild type) or CL2006 (Aβ) worms were applied to SDS-PAGE (Load) or processed as described in Figure 5A (Eluate) before being applied to SDS-PAGE. N2 (wild type) worms were used at day 1 of adulthood, whereas CL2006 (Aβ) worms were used at days 1, 5 or 8 of adulthood. The gel was transferred to nitrocellulose membrane that was probed for Aβ (≈4 kDa) using the 6E10 antibody or for tubulin (≈55 kDa) as a loading control. The amount of sample applied to the gel was 10 fold higher for the eluate (10X) when compared with the load (1X). In lane 9, synthetic Aβ_1–40_ peptide (2 ng) was used as a standard for Aβ. Note that the peptide Aβ_1–40_ runs faster than the Aβ synthesized in the CL2006 worms. (B) Quantification of Aβ bands of panel (A). Since the eluate fractions do not contain tubulin, we normalized the eluate bands using the tubulin bands of the load samples. The quantification was made using Fiji software and the bars represent the standard deviation of two experiments.

Since the IP approach was efficient at pulling down exogenously added Aβ_1–40_ fibrils from wild type worm PDS (Figure 5B), we investigated a more physiological condition using tissue homogenates from a *C. elegans* AD model. The CL2006 strain of *C. elegans* constitutively expresses human Aβ_1–42_ [28] but due to a post-transcriptional modification, this worm accumulates Aβ_3–42_ aggregates [53], [54]. The aggregates of Aβ_3–42_ are present in plaques of brains of patients with AD and the Aβ_3–42_ peptide recapitulates *in vitro* and *in vivo* the amyloidogenic characteristic of the Aβ_1–42_ peptide. As a negative control, we used N2 wild type worm PDS that we used in the previous experiments (Figures 3 and 5) but now in the absence of any added recombinant amyloid fibrils. We divided the samples into two parts: Load, that is, the sample before PK digestion, acetone precipitation and IP using the LOC antibody, and Eluate, the sample after all the aforementioned steps. Again, on the SDS-PAGE gel, we applied 10 fold more of the Eluate fraction (10X) when compared with the Load fraction (1X). After SDS-PAGE, the gel was transferred to a nitrocellulose membrane that was probed with antibodies against Aβ (≈4 kDa) and the housekeeping protein tubulin (≈55 kDa). Firstly, we confirmed that only the CL2006 worms express the Aβ peptide (compare the band around 4 kDa in lane 1 from the N2 wild type worm PDS with that in lane 3 from CL2006 worm PDS). We observed the efficient capture of amyloid fibrils from the CL2006 strain of *C. elegans* using the protocol described in the Figure 5A (Figure 6A lane 4). Since the protocol was able to immunoprecipitate amyloid fibrils produced *in vivo*, we asked whether we could see any difference in the amount of Aβ in the CL2006 strain as the worms age. To address this question, we cultivated the CL2006 worms for 1, 5 and 8 days in the adulthood stage of development. Surprisingly, we observed a similar amount of total Aβ during the aging of the CL2006 worms (compare lanes 3, 5 and 7 of Figure 6A and Figure 6B). In contrast, using the IP strategy described here, we were able to immunoprecipitate a higher amount of Aβ fibrils in older worms (day 8, lane 8 Figure 6B) when compared with the younger ones (days 1 and 5, lanes 4 and 6 of Figure 6, respectively). Interestingly, it is just after 8 days of adulthood (growing at 20°C, the same conditions used herein) that CL2006 starts to exhibit the phenotypic paralysis caused by the proteotoxicity of Aβ expression [55]. We noted that the dimeric band of Aβ was under-recovered in the immunoprecipitated samples when compared with the input (e.g., compare lane 7 with lane 8 of Figure 6A at ≈8 kDa). Since the buffer used to elute the samples from the beads is acidic, we believe that the combination of SDS, 4 M urea, low pH and boiling might be enough to disrupt the fibrils into monomers, resulting in a low amount of dimers. We conclude that our strategy was useful for immunoprecipitating Aβ amyloid fibrils produced *in vitro* (Figure 5B) and *in vivo* (Figure 6) and that by the use of this approach we detected more aggregates in older worms when compared to young worms.

**Figure 7:**
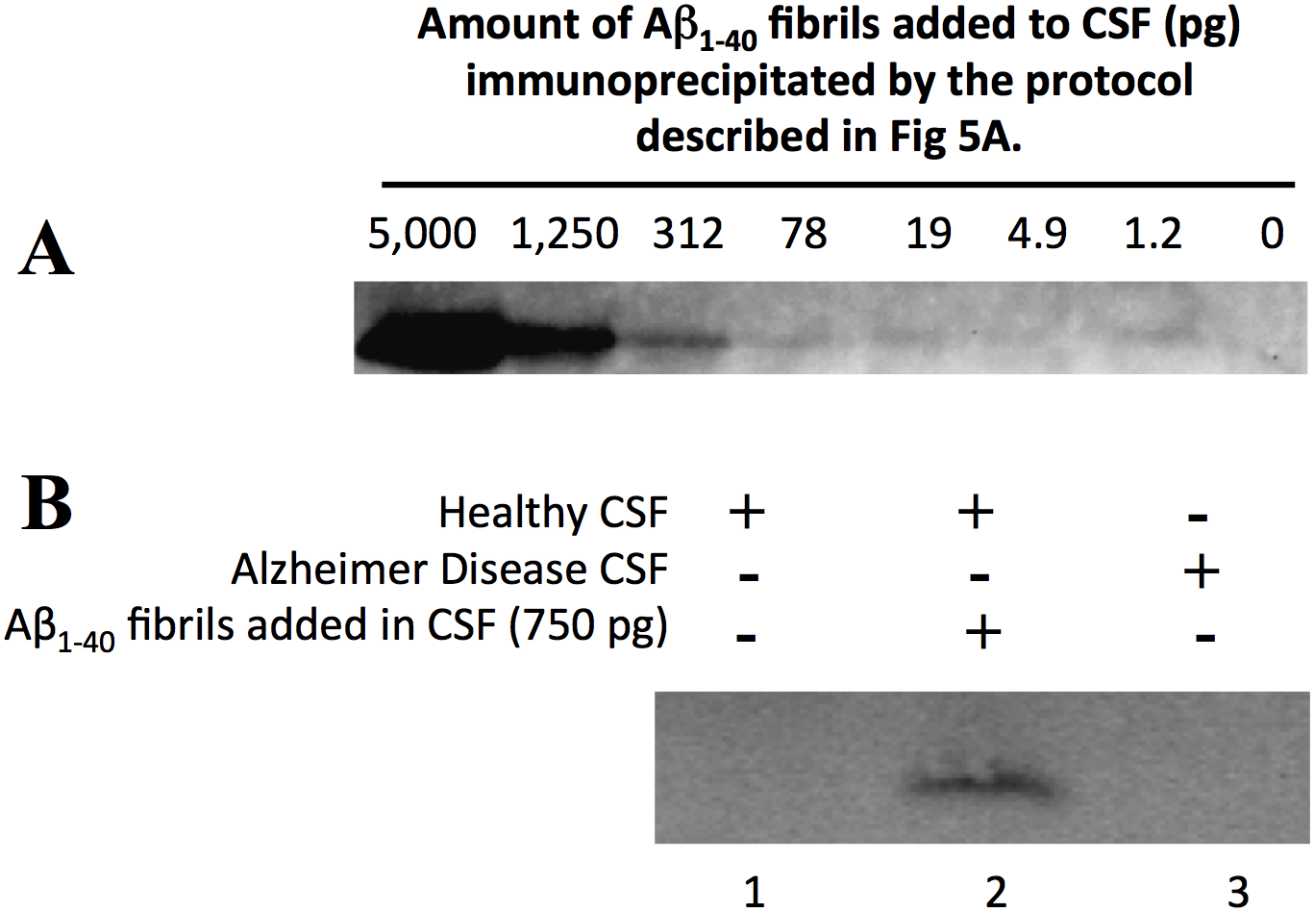
Use of the IP protocol to detect Aβ amyloid fibrils in cerebrospinal fluid (CSF). (A) Different amounts of Aβ_1–40_ amyloid fibrils were added to human CSF and the samples were processed as described in the schematic of Figure 5A. As observed by western blot using the Aβ antibody 6E10, picograms of Aβ fibrils were detected in the eluted fraction of the immunoprecipitated sample. (B) The same experiment described in the panel A was conducted with CSF from patients diagnosed with Alzheimer’s disease and the respective age-matched control. A representative example of one of three CSF samples tested is shown. As positive control, we spiked 750 pg of Aβ_1–40_ amyloid fibrils into human CSF from healthy controls. The absence of detection of soluble Aβ in the CSF is due to its digestion by PK.

Biomarkers are being sought to enable the identification of Alzheimer’s disease before the onset of cognitive dysfunction [56]. Among the potential biomarkers for AD is the detection of oligomeric or fibrillar Aβ species in the cerebrospinal fluid (CSF) of AD patients [56], [57]. As the IP protocol was efficient in detecting Aβ amyloid fibrils produced *in vitro* and *in vivo*, we investigated whether our method could isolate Aβ amyloid fibrils from CSF collected from patients with AD. As a positive control, we spiked different amounts of synthetic pre-formed Aβ_1–40_ fibrils into CSF from healthy controls. As a negative control, we used healthy control CSF. We observed that endogenous Aβ present in CSF, which we were able to detect by western blotting, was completely digested by PK (data not shown). Since the LOC antibody reacts weakly with monomeric Aβ (Figure 4A), PK digestion is essential to ensure that any Aβ detected after IP comes from Aβ fibrils and not from monomeric Aβ. As observed in Figure 7A, immunoprecipitation using the LOC antibody was able to capture and detect 78 pg Aβ_1–40_ fibrils (≈90 pM) spiked into healthy human CSF. This amount represents approximately 0.00002% of the total protein content in CSF [58]. However, we could not detect any Aβ fibrils using the LOC IP protocol in CSF from either healthy controls or AD patients (Figure 7B, lines 1 and 3, respectively). We also omitted the proteinase K and acetone precipitation step before the IP with the LOC antibody but we did not detect any Aβ fibrils from either healthy controls or AD patients (data not shown). To date, Aβ fibrils have been detected in CSF of AD patients in only one study [59]. In this elegant study by Pitschke and colleagues, fluorescently labeled Aβ_1–42_ monomers were added to CSF of AD patients and the presence of large peaks detected by fluorescence correlation spectroscopy indicated polymerization of the fluorescent Aβ_1–42_, seeded by Aβ multimers present in the CSF. These peaks were absent or in lower frequency in the CSF of healthy controls. The linearity of this approach was tested using synthetic Aβ multimers, as in our study, and shown to be between 1–50 µg/ml, 3 orders of magnitude less sensitive than our protocol. A plausible explanation for this discrepancy might be due to the different methodological approaches used by our group and Pitschke’s group. Recently, we demonstrated that the mechanism of Aβ aggregation is a nucleated conformational conversion mechanism [60] similar to that observed by the yeast prion protein Sup35 [61], [62]. In this mechanism, the protein aggregates from oligomers that are kinetically competent to form amyloid fibrils [60]. Since Aβ oligomeric species have been detected in CSF from AD patients by the use of different approaches [63]–[66], the detection of seeding-competent oligomers instead of mature amyloid fibrils by Pitschke’s group cannot be discarded.

## Conclusions

Several groups have described the use of bioinformatics [67]–[69] and *in vivo* screening [70], [71] to find new amyloid. Biochemical analytical methods are useful for this purpose but few or no targets were subsequently validated by other assays, showing that the isolation of amyloid fibrils is challenging [72]. Our two-step strategy lays the groundwork for developing a sensitive assay for the purification and detection of amyloid fibrils. One limitation of our strategy was the IP step, probably imposed by the affinity of the LOC antibody for α-syn and gelsolin amyloid fibrils. It is important to emphasize that the LOC antibody was able to recognize all amyloid fibrils tested as presented before by Glabe’s group [10]. However, probably due the complexity of the reaction medium used in this work, the ability of LOC antibody to immunoprecipitate different kinds of fibrils was compromised. Nevertheless, we could immunoprecipitate picograms of Aβ fibrils by the use of the protocol described here. We envision a scenario where new amyloid conformational antibodies can be created, making the use of this methodology generic and not restricted to purification of Aβ fibrils. The LOC antibody was efficient in imunoprecipitating Aβ fibrils produced *in vivo* and methodology described here can be useful to purify Aβ fibrils from biological samples, rendering the fibrils available for more accurate structural and biochemical characterization. We hope that the goals and limitations presented in this work give new insight to the research community to enable the development of a method that can be used to isolate amyloid fibrils from complex solutions.
